# Caregiver Stress: Biomarkers Linked to Disease Risk and the Psychobiology of Stress Reduction

**DOI:** 10.1093/geroni/igab046.676

**Published:** 2021-12-17

**Authors:** Janelle Beadle, Felipe Jain

## Abstract

Caregivers to older adults with chronic diseases frequently experience chronic stress which can negatively affect caregivers’ physical and mental health, and increase disease risk. This interdisciplinary symposium will highlight critical factors influencing caregiver stress, and the role of biomarkers in detecting caregiver disease risk. First, we will discuss the effects of stress and emotional experiences on risk for cardiovascular disease in caregivers of persons with dementia (PWD). In the first talk, Dr. Mausbach will examine relationships among perceived stress, blood glucose and risk of diabetes and cardiovascular disease in caregivers of PWD. Next, Dr. Losada-Baltar will discuss the degree to which caregivers’ ambivalent feelings towards providing care are associated with inflammatory markers of cardiovascular risk. Following this, two talks will investigate critical links between stress and caregiver emotional well-being. Dr. Liu will report relationships among the stress-related hormone cortisol, sleep, and anxiety in the context of adult day services. Dr. Beadle will examine the degree to which caregivers’ affiliative, empathetic interactions with others relate to their experience of stress through cortisol assessments and neuroimaging. The final talk by Dr. Jain will investigate the effects of a Mentalizing Imagery Therapy intervention for family PWD caregivers on stress, evidence for mindfulness as a causal mediator of stress reduction, and the relationship to brain networks associated with emotion regulation. Taken together, this symposium will identify relevant psychosocial and biological factors that contribute to caregiver stress, as well as discuss the psychobiology of amelioration of caregiver stress.

